# A novel study on the reduction of non-exhaust particulate matter emissions through system vibration control

**DOI:** 10.1038/s41598-022-11703-w

**Published:** 2022-05-06

**Authors:** Priyadarshini Jayashree, Emiliano Rustighi, Giovanni Straffelini

**Affiliations:** Present Address: Department of Industrial Engineering, University of Trento, Via Sommarive 9, Trento, Italy

**Keywords:** Environmental sciences, Energy science and technology, Materials science

## Abstract

The need to reduce non-exhaust particulate matter emissions is of paramount importance as they pose repercussions on human lives and the environment. In this study, a novel way to limit emissions is proposed based on the minimization of the vibration of the mating bodies. Two model friction material formulations were tested in the form of pins and paired with a pearlitic grey cast iron disc counterface in a laboratory pin on disc apparatus. To reduce the vibrations, a damping tape was wrapped around the pins. With the damping of vibration, a significant drop in the emissions was recorded, and this was correlated with the friction layer establishment during sliding, which observed low disruption. It is believed that the use of this method for reducing emissions can accompany the optimization phase of the brake squeal noise of friction materials, thereby, providing new design perspectives.

## Introduction

During a typical braking process, 40–50% of wear debris generated from the mating interfaces is converted into airborne particulate matter (PM) and released into the atmosphere^1–3^. This contributes to air pollution, which claims over half a million adult lives prematurely every year in the European region^4^. The brake wear PM is mainly categorized based on size. Different fractions of PM have their health repercussions. PM_10_ can be easily inhaled and consists of particles that can be introduced into the body through breathing either by nose or mouth. PM_2.5_ can enter the lungs, PM_1_ reaches the lung alveoli, and ultrafine particles like PM_0.1_ can surpass the air-blood barrier in the alveoli and enter internal organs, including the brain^4–6^.

Based on the different tribological roles, the constituents of friction material formulations for automotive applications can be essentially categorized into binders, reinforcements, fillers, and friction modifiers (abrasives and lubricants)^7^. In terms of friction and wear, the brake performances are related to the characteristics of the friction layer formed on the mating surfaces during sliding. The friction layer is formed by primary plateaus, typically made of reinforcements (such as the steel wools) or large abrasives, against which wear fragments are compacted, forming the secondary plateaus^8,9^. Wear particles, including the emitted airborne particles, are generally a result of the disruption of the secondary plateaus^10–14^. As far as the emission evaluation is concerned, by following a pre-defined protocol^13,15^, a ‘model scenario’ of PM emissions can be achieved through a pin on disc (PoD) tribometer, as it can be completely enclosed and its ‘environment’ can be easily controlled^3^.

There are many inherent factors like composition, coupling properties, and working conditions that could contribute towards the removal of the secondary plateaus, thereby, increasing the emission concentration. This study reveals a one-of-a-kind relationship between emissions and system vibrations. The concept of noise, vibration, and harshness (NVH) is usually studied to enhance vehicle longevity and customer comfort^16^. However, to the best of the Authors’ knowledge, the concept of relating emissions with system vibration is relatively new and calls for further study.

The emission-vibration relationship is first investigated using two ‘in-house’ formulated friction material compositions, through a specific apparatus setup attached to the PoD. Subsequently, a possible solution to lower the emissions by reducing the vibration of the medium through damping using a thin rubber tape is proposed and investigated. The comparison is also extended to the friction coefficient (CoF), pin wear evaluation, and the coverage and characteristics of the deposited secondary contact plateaus in both cases. Finally, a possible explanation and solution to reduce the PM emissions are highlighted for newly developed friction material formulations intended to be employed in automotive braking applications.

## Results

### Testing method

Two ‘in-house’ formulated friction material compositions were analyzed to obtain the vibration–emission relationship. The formulations were named basic composition (BC) and modified composition (MC). The compositions had different steel wool content (22 wt.% in BC and 5 wt.% in MC) to induce different adhesive interactions with the cast-iron disc counterface^17–19^. The vibration–emission relationship was obtained by dry sliding tests on a PoD tribometer at a contact pressure of 1 MPa, sliding velocity of 1.51 m/s, testing duration of 90 min, and at room temperature testing conditions. These parameters correspond to mild braking conditions, characterized by the prevailing emission of fine particles. The ultrafine particles offer only a small contribution due to the rather low contact temperatures^8,14^. The fine particle concentration (emissions) was obtained from an Optical Particle Sizer Spectrometer (OPS) and the vibration measurements (RMS) was obtained by fixing two shear accelerometers at different locations on the pin holder (named A1 and A2). On the other hand, the tests with damping were conducted on the same two friction material compositions and at same testing conditions, but by wrapping them around with a rubber tape (thickness: 0.75 mm, material: low-density polyethylene, LDPE). Care was taken to cover the sides and the top surface of the pin, and the analysis was conducted at the same testing conditions mentioned previously.

### Relationship between emissions and vibrations

**Figure 1:**
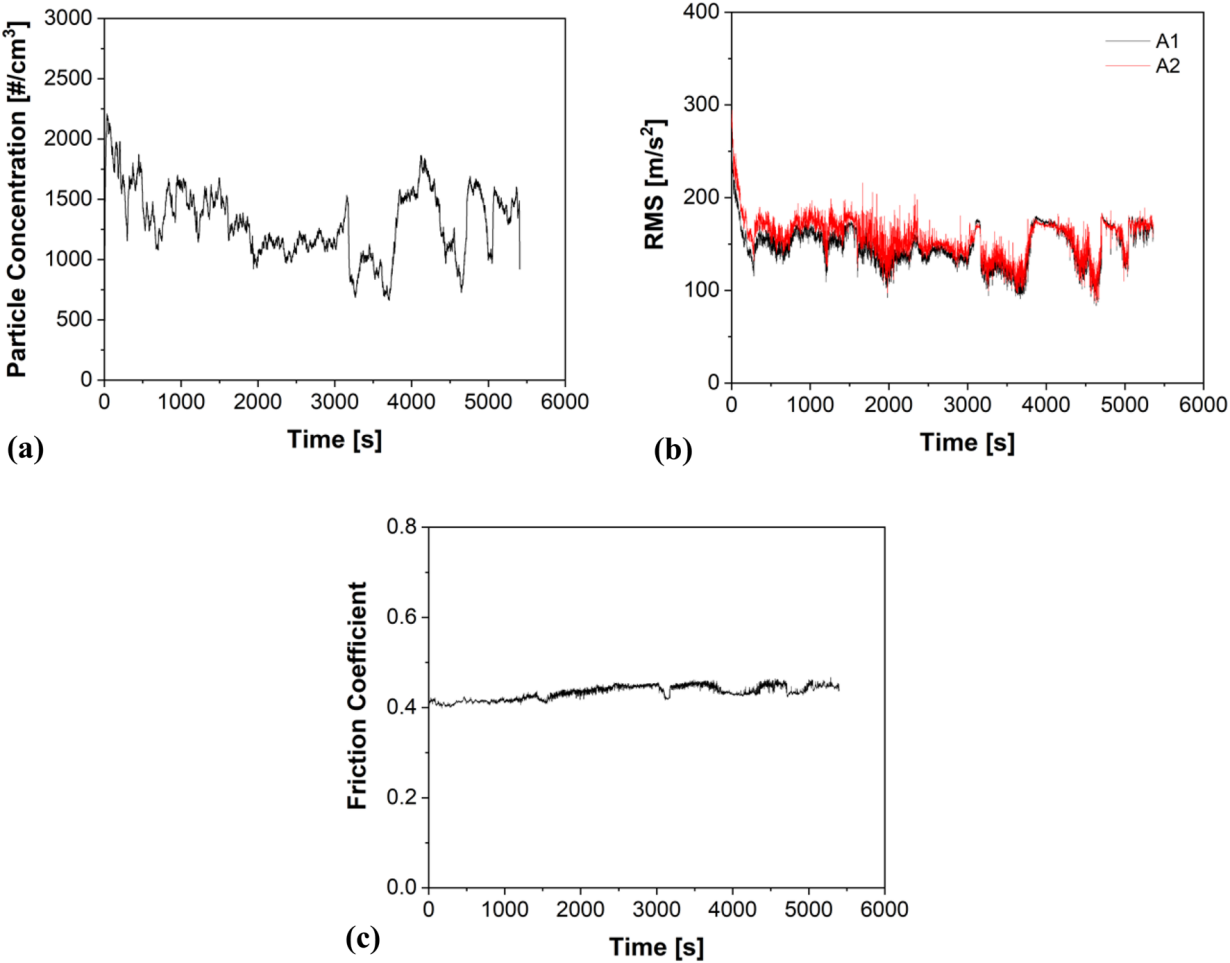
A typical example of (**a**) Emissions; (**b**) Vibrations; and (**c**) CoF trends demonstrated by MC specimens.

As a typical example, Fig. 1 presents the particle concentration, vibration, and friction coefficient (CoF) trends of MC. From Fig. 1a,b, a clear relationship between the emission and vibration curves can be appreciated. Each rise and fall of the curves are mimicked by both traces to a certain level of precision. Figure 1b shows the similar vibration RMS magnitude of shear accelerometers A1 and A2. Figure 1c shows the CoF curves and trends.

### Reduction of vibration through damping tape

**Figure 2:**
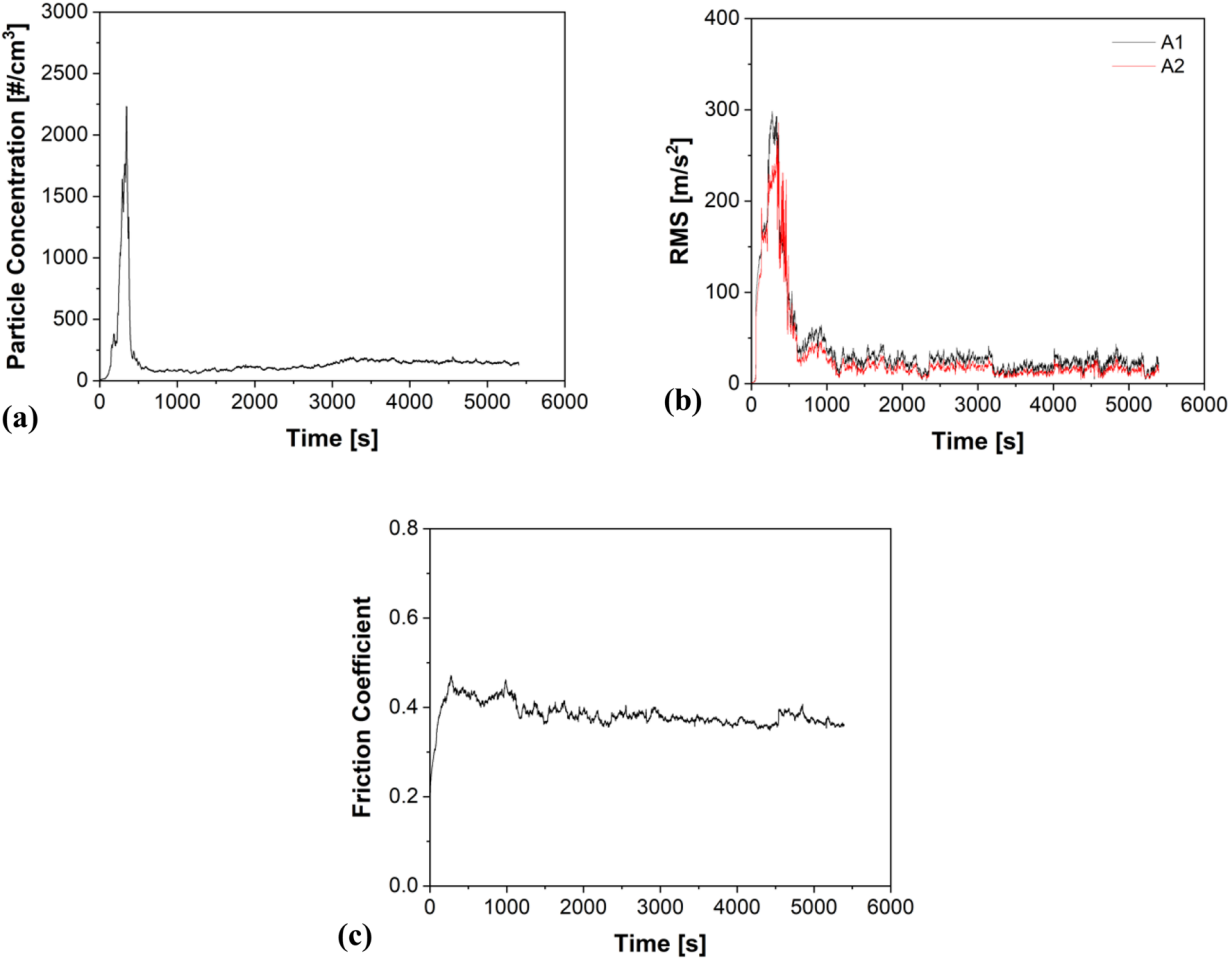
A typical example of (**a**) Emissions; (**b**) Vibrations; and (**c**) CoF trends demonstrated by MC specimens with the damping tape.

As mentioned previously, the second category of tests with the friction materials was conducted by wrapping the pins and covering their top surfaces with a thin rubber tape. Figure 2 shows a representative example of the analysis with the rubber tape with MC. Firstly, like Fig. 1a,b, a concrete relationship between emissions and vibrations can be observed. Secondly, from Fig. 2a,b, a drastic drop in particle concentration and vibrations are noted, obtaining a steady state after a running-in stage of approx. 2000s.

**Figure 3:**
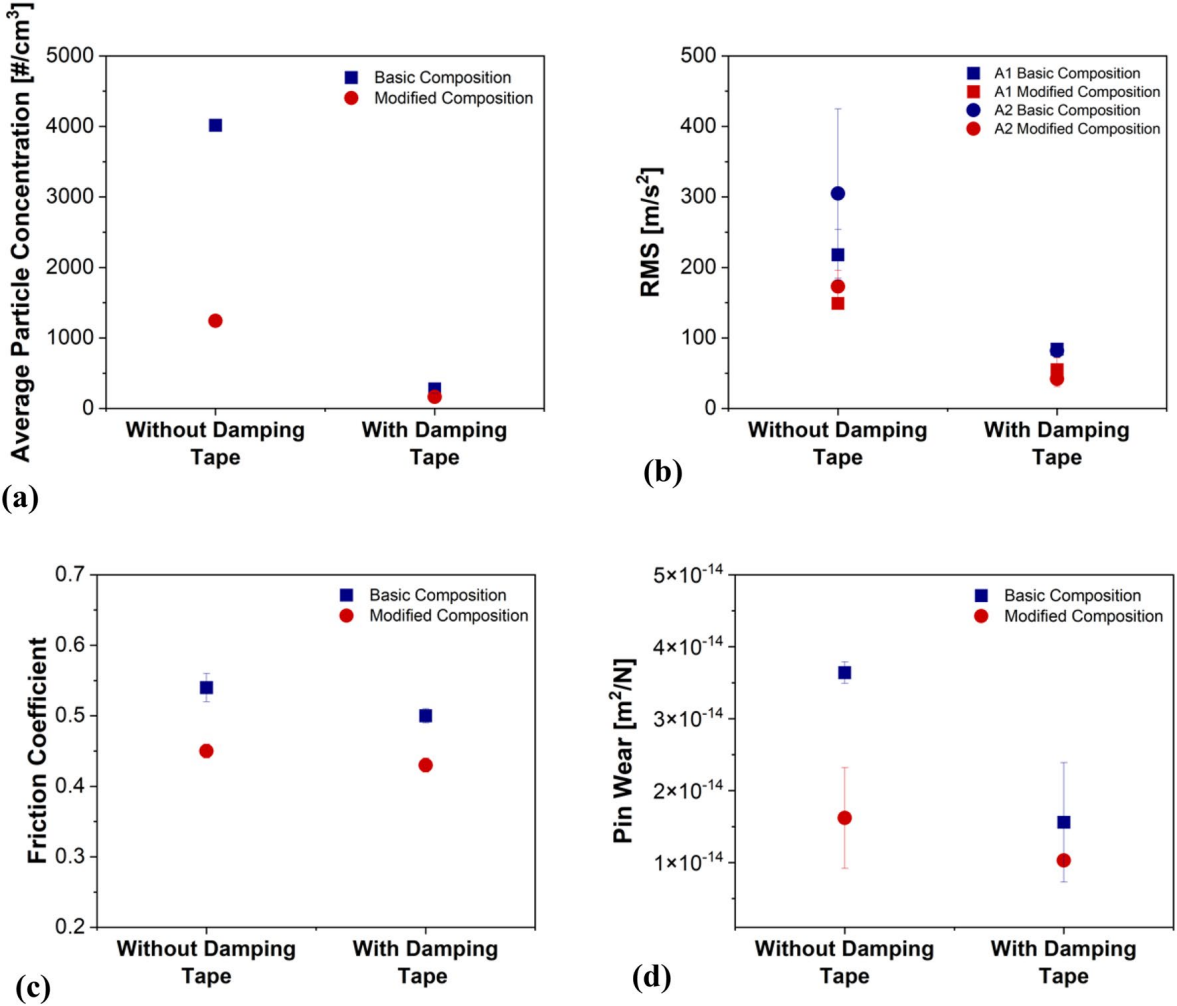
Comparison of (**a**) Emission; (**b**) Vibration (A1 and A2); (**c**) CoF trends; and (**d**) Pin wear with and without damping tape for the BC and MC specimens.

Figure 3 presents the comparison of emissions, vibrations, CoF, and pin wear magnitude of BC and MC with and without damping tape (average values are considered, obtained after the run-in period). As a general trend, irrespective of the presence of the damping tape, the emissions, vibrations, CoF, and pin wear are seen to decrease when moving from BC to MC. With the utilization of the damping tape, irrespective of the composition, the average particle concentration, vibrations recorded with both accelerometers, and the pin wear is observed to reduce significantly when compared to the trials without the damping tape. Alternatively, only a slight dip in the steady-state CoF magnitude is observed with the presence of damping tape for both BC and MC.

### Worn surface characteristics

**Figure 4:**
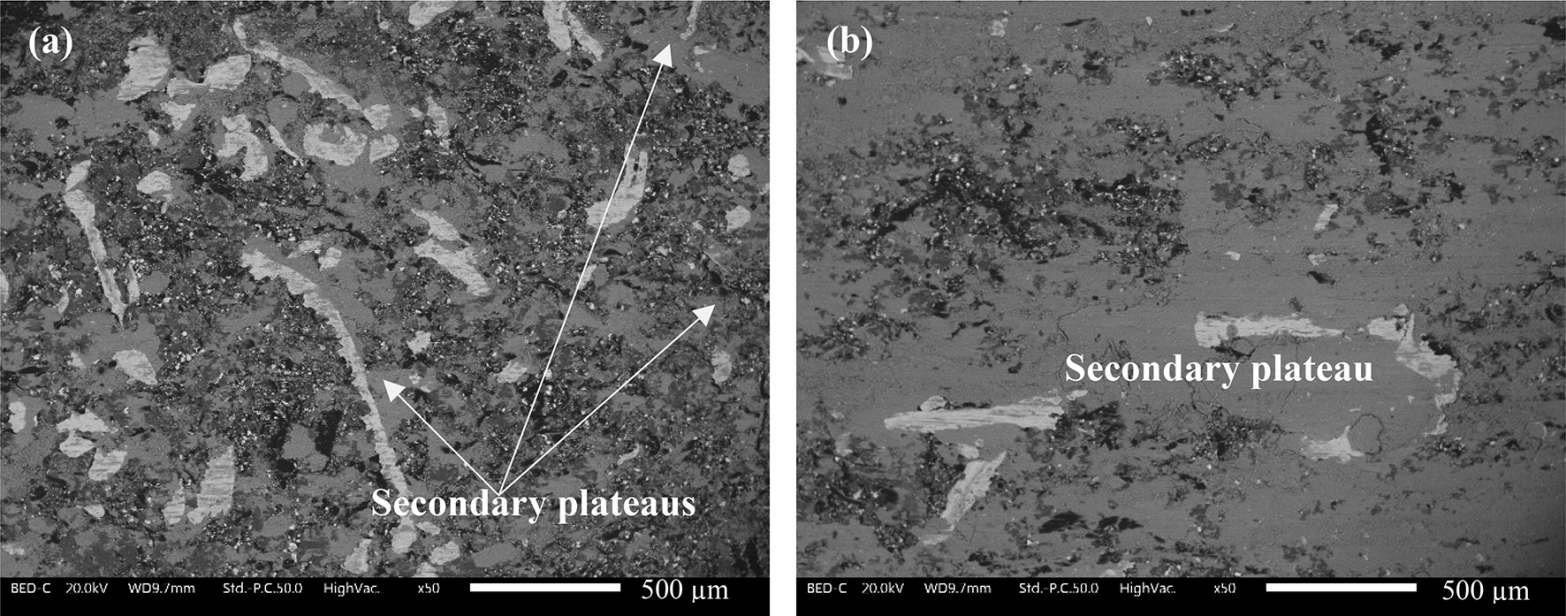
Typical examples of worn pin surface (**a**) Without damping tape; and (**b**) With damping tape in the BC specimens.

Figure 4 shows the worn surfaces of a BC pin without (Fig. 4a) and with damping tape (Fig. 4b). In Fig. 4a, the worn surface is covered by white steel fibers. In their vicinity, compacted light grey islands made of Fe oxides are present, known as the secondary contact plateaus. The extension of the secondary plateaus is quite limited and is surrounding the steel fibers. In contrast, Fig. 4b shows a highly extended, extremely compacted, and smooth secondary plateau. Here, the presence of steel fibers on the surface is quite low and is partially covered by the secondary contact plateaus, which are predominantly made of Fe oxides.

**Figure 5:**
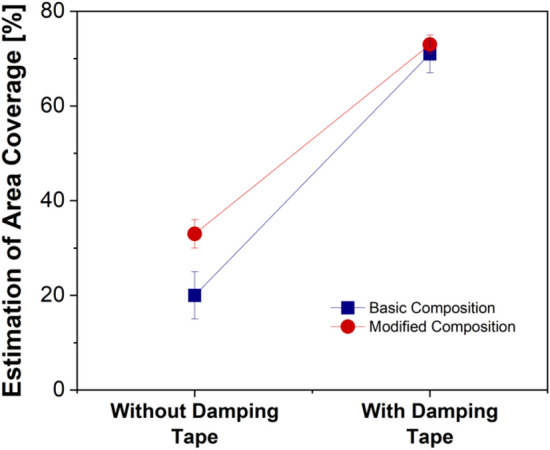
Comparison of the estimation of secondary plateau area coverage without and with damping tape for the BC and MC specimens.

To further understand the extension of the secondary plateaus deposited on worn pin surfaces with and without the damping tape, a basic estimation of secondary plateau area coverage was conducted using the ImageJ open-source software. Figure 5 displays the secondary plateau coverage for BC and MC with and without damping tape. In both cases (BC and MC), the area coverage of the secondary plateaus is observed to increase significantly with the presence of the damping tape. The highest increase is observed for the BC specimens. Nevertheless, with damping, the BC and MC specimens have similar coverage (71 and 73% respectively).

## Discussion

As seen from Fig. 4a, the BC has a significant presence of steel fibers on the worn pin surface. Maatta et al.^20^ have explained that the Fe–Fe interaction (in this case, steel fibers–cast-iron disc counterface) leads to strong adhesion, resulting in comparatively higher CoF and friction induced vibration^17^. Essentially, from the Fe–Fe adhesion and the relative motion between the pin and disc, the vibration of the pin holder is observed (Fig. 3b). The pin holder vibration creates continuous disturbance between the pin–disc interface, leading to the routine disruption of the secondary contact plateaus, which is presented by Fig. 4a, wherein, the limited extension of the secondary contact plateaus can be observed. Numerous studies^13–15^ have shown that the predominant PM constituents are detached secondary plateaus. With the high removal of the secondary plateaus in the BC specimens, a naturally high emission magnitude of fine particles (Fig. 3a) was observed. Furthermore, the secondary contact plateaus also protect the pin from high wear. In their absence, the BC observed elevated pin wear (Fig. 3d). The MC inherently has low steel fiber content (5%, Table 1), and the Fe powder (5%, Table 1) is dispersed in the bulk, which reduces the adhesion and the subsequent hard effects of shearing action on the system, leading to lower CoF and vibrations (Fig. 3b,c). The reduced vibrations also resulted in lower emissions (Fig. 3a) and pin wear (Fig. 3d), when compared to the BC specimens.

**Table 1:** BC and MC composition (in wt.%).

Composition	Binder	Graphite	Barite + calcite	Steel wool	Aramid fibers	Alumina	Tin sulfide	Vermiculite	Iron powder
BC	8	15	20	22	5	30	–	–	–
MC	8	10	25	5	7	20	10	10	5

The presence of a thin rubber tape effectively dissipated the energy associated with the generated vibration^21^. This is shown by the reduction in the RMS measurements in Fig. 3b for both compositions. With the reduction in the pin holder vibration, the situation was ideal for the formation and sustenance of smooth, compacted, and extended secondary contact plateaus, as shown in Figs. 4b and 5. With the presence of a high-quality secondary plateau (as shown by the comparatively high extension in Fig. 5), both BC and MC observed significantly lower emissions and pin wear (Fig. 3b,d). In previous studies^22,23^, it was shown that the CoF of a system is dependent on the constituents of the interacting surfaces. The damping tape and the subsequent formation of a highly extended and thick secondary contact plateau avoided the Fe–Fe interaction, resulting in slightly lower CoF (Fig. 3c). At this point, an interesting observation is the similar average particle concentration (Fig. 3a), vibrations (Fig. 3b), and secondary plateau extension (Fig. 5) for both BC and MC with the damping tape. This infers that irrespective of the composition, with effective damping, any kind of formulation could achieve low PM emissions. The future of this study will be focused on the evaluation of commercial friction material formulations for automotive braking applications.

## Methods

### Materials

Two types of friction material compositions, formulated in-house, were evaluated. These compositions constituted of only essential constituents and were utilized for evaluating the properties of additives (like newly developed abrasives, lubricants, or fillers). The compositions were titled ‘basic composition’ (BC) and ‘modified composition’ (MC), and the constituents are given in Table 1.

The two formulations (BC and MC) were tested in the form of pins. The pins were obtained through a pre-defined production procedure^24,25^. All the constituents in Table 1 were carefully weighed, and except for the steel wool, were mixed continuously in a TURBULA^®^ mixer for 20 min. After the initial mixing, the steel wool was then added to the mixture and mixed for an additional 10 min. The extra step of separate steel wool addition and subsequent mixing was incorporated, as it would avoid any clumping and agglomeration of steel wool, which occurs at longer mixing duration. The well-mixed powders were subjected to the hot-pressing procedure to obtain pins. The required quantity of powder was taken and tap-pressed into a tool steel cylindrical mold of hot-pressing equipment (BUEHLER^®^ hot mounting press) at a pressure of 100 MPa, temperature of 150 °C, and holding time of 10 min. Lastly, the produced green body was treated to a post-curing process at 200 °C for 4 h. All pin compositions had an average height and diameter of 10 mm. The average density obtained from 10 pins were: BC: 2.75 g/cm^3^ and MC: 2.50 g/cm^3^. The pins were paired with a pearlitic grey cast iron counterface in the form of discs (diameter: 60 mm; thickness: 6 mm).

### Experimental setup and testing conditions

**Figure 6:**
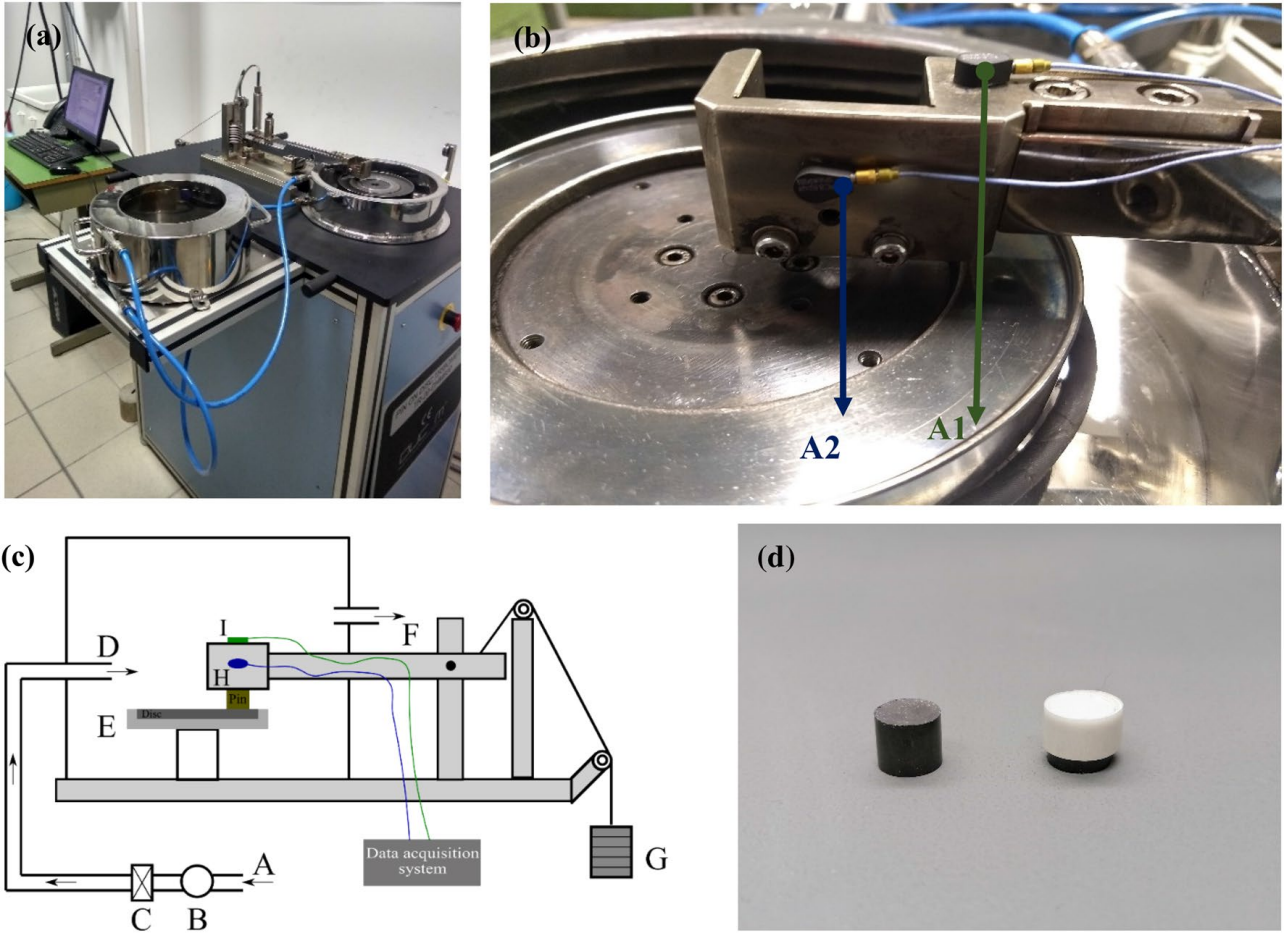
(**a**) PoD tribometer; (**b**) Shear accelerometer positions; (**c**) Testing apparatus setup (A) Ambient air, (B) Fan, (C) HEPA filter, (D) Air introduced in the chamber, (E) Disc/Counterface, (F) Air outlet to the OPS, (G) Weights, (H) & (I) Shear accelerometers attached to the pin holder; (**d**) A typical example of pins with and without the rubber tape.

The dry sliding tests and the corresponding emission and vibration analysis were conducted on a pin on a disc (PoD) tribometer. Figure 6 shows the experimental setup of the analysis. Figure 6a presents the PoD equipment, with Fig. 6b showing the placement of the shear accelerometer sensors on the pin holder arm in two different locations named A1 and A2. The nominal sensitivity of A1 and A2 were 9.89 and 9.77 mVm/s^2^ respectively. The friction coefficient was obtained directly from the software linked to the PoD equipment. The shear accelerometers were connected to a DEWESoft–SIRIUSm data acquisition system, shown in Fig. 6c. The data acquisition system mainly recorded RMS of the acceleration at a sampling rate of 20,000 samples/s. Figure 6c also shows the schematics for particle collection attachment setup. The air from the laboratory (A) is pulled in using a fan (B), which is circulated in a High-Efficiency Particulate Air (HEPA) filter (C) to remove any dust speckles and impurities, thereby, resulting in purified air (particle concentration maintained below 10 #/cm^3^) being introduced in the PoD chamber (D). The air velocity was maintained at 11.5 m/s^13^. To obtain particle concentration, a TSI^®^ (TSI Incorporated, Shoreview, USA) Optical Particle Sizer Spectrometer (OPS, model 3330) was connected to the PoD tribometer at Site F in Fig. 6c. The OPS typically measures particle concentration in the size range from 0.3 to 10 μm, split into 16 channels, and with a sampling frequency of 1 Hz. The OPS operates with a self-controlled sampling flow rate of 1 l/min. Like the CoF, the particle concentration was directly obtained from the software connected to the OPS. The specific wear coefficient (pin wear) was calculated by weighing the pins before and after each trial, and calculated from the equation:

$${\text{K}}_{{\text{a}}} = \frac{V}{{\left( {F \times d} \right)}}$$

where: *V*: wear volume loss; *F*: load applied; *d*: sliding distance (~ 8150 m).

The trials were conducted at a contact pressure of 1 MPa (79 N), sliding distance of 1.51 m/s (for a wear track of 48 mm, amounted to 600 rpm), 90 min of testing duration, and at room temperature testing condition (23–25 °C). Prior to the beginning of any tests, all combinations of pins and discs were subjected to 30 min long ‘run-in’ procedure (at the aforementioned testing conditions) to achieve a conformance between the mating surfaces of the pins and the discs. The humidity of the lab was not controlled but was regularly monitored, varying in the range of 40–45%. To reduce the effect of humidity variation, all trials and corresponding repetitions were conducted within a span of 10 days. Four trials were conducted for each composition at different testing conditions (with and without damping). An example of pins with and without the rubber tape are shown in Fig. 6d.

### Characterization of worn surface

The worn surfaces of the pins were evaluated using a scanning electron microscope (SEM, Make: JEOL IT300). A basic estimation of the area of secondary plateau coverage on the worn pin surfaces of BC and MC specimens with and without damping were evaluated using the ImageJ open-source software. The evaluation was conducted on ten different sites from multiple specimens at 50 × magnification.

## Data Availability

The data generated during and/or analyzed during the study are available from the corresponding author up on request.
